# Ultrasonic Characteristics Improve Prediction of Central Lymph Node Metastasis in cN0 Unifocal Papillary Thyroid Cancer

**DOI:** 10.3389/fendo.2022.870813

**Published:** 2022-06-20

**Authors:** Yongchen Liu, Jianhao Huang, Zhiyuan Zhang, Yijie Huang, Jialin Du, Sanming Wang, Zeyu Wu

**Affiliations:** ^1^ Department of Thyroid and Hernia Surgery, Guangdong Provincial People’s Hospital, Guangdong Academy of Medical Sciences, Guangzhou, China; ^2^ Shantou University Medical College, Shantou, China; ^3^ The Second School of Clinical Medicine, Southern Medical University, Guangzhou, China

**Keywords:** ultrasonic characteristics, CLNM, cN0 PTC, unifocal, predictor

## Abstract

**Background:**

Prediction of central lymph node metastasis (CLNM) is vital for clinical decision-making processes in clinically N0 (cN0) unifocal papillary thyroid carcinoma (PTC), but the sensitivity of preoperative detection of CLNM is limited. The aim of the present study was to determine whether there are ultrasonic (US) characteristics associated with CLNM.

**Methods:**

In total, 1657 PTC patients (514 men and 1143 women) were enrolled in the present study between January 2018 and May 2021. The patients met the following inclusion criteria based on preoperative detection: suspected nodule confirmed as PTC by biopsy; the nodule was unifocal and less than 4 cm in diameter; no prior neck radiation exposure; no extrathyroidal extension; and no CLNM or distant metastases on imaging. All the enrolled patients underwent total thyroidectomy with prophylactic central lymph node dissection (CLND). A postoperative pathological diagnosis was made.

**Results:**

CLNM was found in 58.4% of male patients and 36.9% of female patients. In univariate analysis, size, adjacent anterior capsule, distance to the lower pole and color Doppler flow imaging (CDFI) were considered risk factors for the male and female groups (p < 0.05). In multivariate analyses, size, adjacent anterior capsule, distance to the lower pole and CDFI were independent risk factors for male patients. For females, the independent risk factors included size, adjacent anterior capsule, distance to the lower pole and CDFI.

**Conclusion:**

In the present cohort, US imaging characteristics, including size, adjacent anterior capsule, distance to the lower pole and CDFI, were identified to be potentially beneficial in preoperative clinical decision-making processes for cN0 unifocal PTC patients.

## Introduction

Thyroid carcinoma is one of the most common neoplastic diseases (1), and its morbidity is increasing worldwide (2). The age-standardized incidence of thyroid carcinoma is over 5% in some Asian countries (3), and this carcinoma occurs approximately three times more often in women than in men (4). Papillary thyroid carcinoma (PTC) accounts for approximately 95% of thyroid carcinomas and generally has an excellent prognosis with 10-year survival rates approaching 90-95% (5). Nevertheless, because PTC represents approximately 95% of all cases, most cancer related mortality is due to PTC.

The primary treatment method of PTC is surgical ablation. The objectives of initial surgical therapy include removing the primary tumor and clinically significant cervical lymph nodes as well as minimizing treatment-related morbidity and the risk of recurrence or metastasis. Recent American Thyroid Association (ATA) guidelines and National Comprehensive Cancer Network (NCCN) guidelines note primary risk factors for preoperative determination of the thyroid resection extent (6, 7). Lobectomy is indicated if the following criteria are met: no prior radiation exposure, no cervical lymph node metastases, no extrathyroidal extension, no distant metastases and tumor size less than 4 cm in diameter. For these criteria-matched clinically N0 (cN0) unifocal PTCs, routine prophylactic central compartment lymph node dissection (CLND) is not recommended by both guidelines. However, central compartment lymph node metastasis (CLNM) is relevant to risk stratification and prognosis (8, 9). Currently, there are no non- or minimally-invasive methods that are completely reliable for detecting all of the potential metastases (10). Thus, an accurate preoperative evaluation of CLNM is vital for the management of PTC patients.

For cN0 unifocal PTCs, the accurate identification of CLNM is crucial. Nonetheless, CLNM is difficult to detect preoperatively, and the current assessment methods have limited power. Approximately 30–80% of PTCs are associated with CLNM (11–13), and some studies have suggested that CLNM is related to disease relapse and distant metastases (14–16). As CLNM is difficult to detect preoperatively and CLND is related to morbidity (17), the clinical decisions for treatment are controversial (18). Previous medical studies have suggested that CLND may reduce the recurrence of PTC, indicating a risk stratification for recurrence and distant metastases. In addition, the treatment procedure, such as radioactive iodine therapy, may be altered accordingly. For those criteria-matched cases, prophylactic CLND permits patients to obtain more active medical treatment and less hazardous reoperative surgical treatment (19, 20). However, prophylactic CLND increases the morbidity, such as hypoparathyroidism and recurrent laryngeal nerve injury (21, 22). The ATA and NCCN guidelines do not suggest prophylactic CLND, stating that prophylactic CLND may be considered in specific patients who have advanced primary tumors. There is also a viewpoint that more evidence is needed to support that prophylactic CLND is beneficial to reduce recurrence rates (23). Above all, a more accurate evaluation of CLNM is necessary for cN0 unifocal PTC patients to obtain better clinical decisions.

Ultrasonic (US) detection is the preferred diagnostic method for CNLM. Although it has many advantages, there are limitations. For example, the sensitivity of US detection in evaluating CLNM ranges from 20 to 60% (24–26). Although many studies have reported high-risk factors related to clinical and US characteristics predictive of CLNM of PTC patients, the conclusions are controversial. Some of the identified risk factors, such as tumor differentiation, extrathyroidal invasion and gene type, are only available postoperatively (27), indicating that they cannot provide reliable information for preoperative clinical decision-making processes. Consequently, the research on a noninvasive and valuable approach based on US detection for evaluating CLNM is essential but challenging.

The present study aimed to evaluate the US imaging characteristics of nodules associated with CLNM in cN0 unifocal PTC patients. The present conclusions may be useful in preoperative clinical decision-making processes.

## Materials and Methods

### Patient Data and Ethical Approval

**Figure 1:**
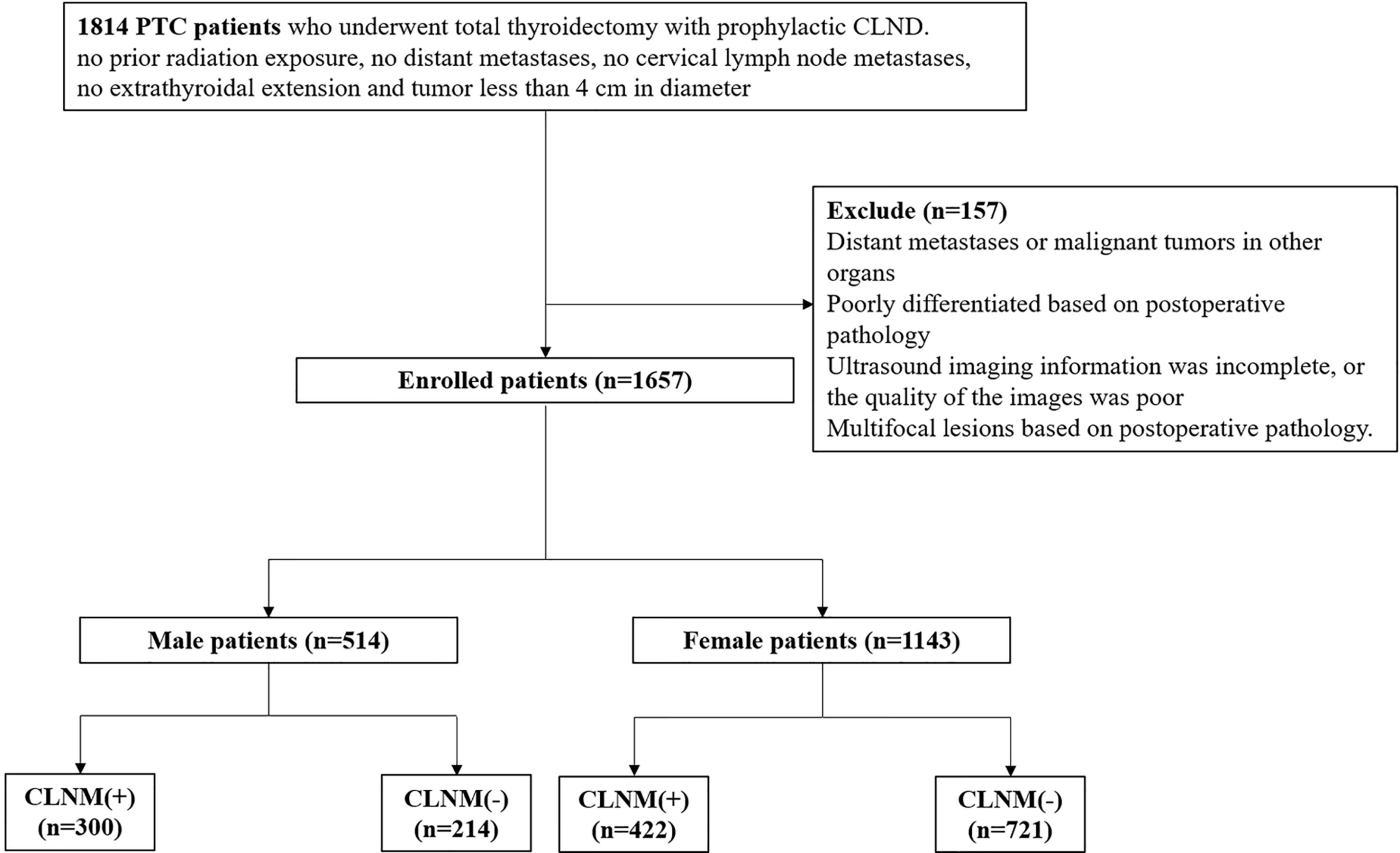
Flow chart of the patients enrolled in our study.

The studies involving human participants were reviewed and approved by the Ethics Committee of the Guangdong Provincial People’s Hospital, Guangdong Academy of Medical Sciences (Guangzhou, Guangdong Province, People’s Republic of China), and they conformed to the provisions of the Declaration of Helsinki. Written informed consent from the patients was not required to participate in this study in accordance with national legislation and the institutional requirements. We evaluated the patients retrospectively with histologically confirmed PTC in our hospital between January 2018 and May 2021. The patients were enrolled according to the following criteria: (1) the suspected thyroid nodule was unifocal and less than 4 cm in diameter based on US examination; (2) the suspected nodule was confirmed to be malignant by ultrasound-guided puncture biopsy; (3) no extrathyroidal extension and no cervical lymph node metastases based on US examination; (4) patients were subjected to an initial thyroid surgery with CLND and were histologically confirmed as having PTC; and (5) no prior neck radiation exposure. Patients were excluded based on the following criteria: (1) having distant metastases or malignant tumors in other organs; (2) poorly differentiated based on postoperative pathology; (3) the US imaging information was incomplete, or the quality of the images was unclear; or (4) multifocal lesions based on postoperative pathology. **Figure 1** shows the patient recruitment process. Ultimately, 1657 patients (514 men and 1143 women) were included in the present study. The data were divided into CLNM-negative and CLNM-positive groups according to the pathology results.

### US Equipment and Evaluation of US Characteristics

US examinations were performed using HI Vision 900, HI Vision Ascendus and HI Vision Preirus color US units (with US elasticity imaging capability) from Hitachi, and the probe frequency was 6.0–13.0 MHz. The US imaging features of every patient were retrospectively re-examined by two independent radiologists with more than 10 years of experience in thyroid US imaging; neither observer knew the clinical nor the pathological outcomes. If the radiologists faced a dilemma, they would determine their final decisions by a consensus. The imaging characteristics of each nodule were as follows: tumor size; multifocality; aspect ratio (height divided by width on transverse views, A/T); tumor location; distance between the nodules and the adjacent capsule; microcalcification situation; border; US halo; tumor internal vascularity; and Hashimoto’s thyroiditis. Many images of the longitudinal and transverse axes were fully evaluated. The tumor size refers to the maximum diameter (D) of the nodule, which was classified as follows: D ≤ 0.5 cm, 0.5 < D ≤ 1.0 cm, 1.0 < D ≤ 1.5 cm and D > 1.5 cm. The A/T was classified as ≤ 1 or > 1. The location of the tumor was evaluated according to the following three aspects: location (left lobe, right lobe and isthmic), distance to the upper pole and distance to the lower pole. The distance between the tumor and adjacent capsule (anterior and posterior) was classified into three categories as follows: < 1 mm and not protruding outside the thyroid capsule; 1 ≤ and < 2 mm; and ≥ 2 mm. Tumor vascularity was classified from 0 to 3 and evaluated by color Doppler flow imaging (CDFI). Hashimoto’s thyroiditis was diagnosed on the basis of US characteristics. Because the diagnostic performance of the present study depended on the accuracy of operator-reported imaging features, the interobserver reproducibility for US features was assessed. Regarding the preoperative identification of cervical lymph nodes (LNs), a LN was considered suspicious if it had one of the following characteristics: microcalcifications, hyperechoic change, loss of fatty hilum; round shape; and necrosis (28).

### Statistical Analysis

Statistical analysis was performed with SPSS Statistics version 24.0 (IBM Corp.). Categorical variables are presented as numbers and percentages. A chi-square test or Fisher’s exact test was used to assess differences between groups. A logistic regression model was used to evaluate the risk factors. The reported statistical significance levels were all two-sided with statistical significance set at 0.05.

## Results

### Characteristics of Patients

Among the 1657 patients, there were 514 (31.0%) male patients and 1143 (69.0%) female patients. A significant difference was found in gender between CLNM-positive and CLNM-negative patients; 58.4% of males and 36.9% of females were CLNM-positive patients (p < 0.05). The gender disparities in incidence, prognosis and aggressiveness are well established for PTC, but the underlying causes remain poorly understood. Population-based studies have shown that reduced estrogen exposure favors PTC malignancy (29, 30). To adjust for the gender factor, we arranged two separate groups for these patients.

### Risk Factors for Male and Female Patients With cN0 Unifocal PTC

The patient features and US imaging characteristics of thyroid nodules in the male and female cohorts are shown in **Tables 1** and **2**. Univariate and multivariate analyses were conducted to determine the differences in clinical and US imaging features between CLNM-positive and CLNM-negative groups. In univariate analysis, size (p < 0.05), adjacent anterior capsule (p < 0.05), distance to the lower pole (p < 0.05) and CDFI (p < 0.05) were considered risk factors for both male and female groups (**Tables 1** and **2**). In multivariate analyses, size, adjacent anterior capsule, distance to the lower pole and CDFI were considered independent risk factors (**Tables 3** and **4**). For male patients, size (0.5-1 cm, OR 2.62, 95% CI 1.18-5.81; 1-1.5 cm, OR 6.93, 95% CI 3.01-15.95; >1.5 cm, OR 12.12, 95% CI 5.21-28.18), adjacent anterior capsule (1-2 mm, OR 2.00, 95% CI 1.32-3.03; <1 mm, OR 2.81, 95% CI 1.83-4.33), distance to the lower pole (10-20 mm, OR 1.52, 95% CI 1.02-2.27; 0-10 mm, OR 2.63, 95% CI 1.69-4.09) and CDFI (2-3, OR 0.69, 95% CI 0.48-1.00) were considered independent risk factors. For female patients, size (0.5-1 cm, OR 0.89, 95% CI 0.53-1.49; 1-1.5 cm, OR 1.43, 95% CI 0.81-2.52; >1.5 cm, OR 4.90, 95% CI 2.73-8.77), adjacent anterior capsule (1-2 mm, OR 3.21, 95% CI 2.21-4.46; <1 mm, OR 5.14, 95% CI 3.60-7.34), distance to the lower pole (10-20 mm, OR 2.06, 95% CI 1.44-2.94; 0-10 mm, OR 3.58, 95% CI 2.36-5.42) and CDFI (2-3, OR 1.82, 95% CI 1.33-2.48) were considered independent risk factors.

**Table 1:** Clinical and US imaging characteristics of male patients.

Characteristics	CLNM		P value
	+	-	
**Total**	300	214	
**Size**			**<** 0.05
0-0.5 cm	8	40	
0.5-1 cm	103	95	
1-1.5 cm	72	41	
> 1.5 cm	117	38	
**Adjacent anterior capsule**			**<** 0.05
< 1 mm	128	25	
1-2 mm	76	60	
≥ mm	96	129	
**Distance to the lower pole**			**<** 0.05
0-10 mm	120	43	
10-20 mm	102	67	
≥ 20 ram	78	104	
**CDFI**			**<** 0.05
02-Mar	129	179	
0-1	171	35	
**Age (year)**			0.27
≥ 55	145	114	
< 55	155	100	
**A/T**			0.83
> 1	171	120	
≥ 1	129	94	
**Location**			0.34
left lobe	127	95	
right lobe	155	100	
Isthmic	18	19	
**Adjacent posterior capsule**			0.17
< 1 mm	76	42	
1-2 mm	89	59	
≥ 2 mm	135	113	
**Distance to the upper pole**			0.9
0-10 mm	76	51	
10-20 mm	103	77	
≥ 20 mm	121	86	
**Microcalcification**			0.09
Present	225	146	
Absent	75	68	
**Smooth border**			0.32
Absent	283	206	
Present	17	8	
**Ultrasonic halo**			0.54
Absent	264	192	
Present	36	22	
**Hypoechoic**			0.34
Present	280	204	
Absent	20	10	
**Hashimoto's thyroiditis**			0.85
Present	17	13	
Absent	283	201	

**Table 2:** Clinical and US imaging characteristics of female patients.

Characteristics	CLNM		P value
	+	-
**Total**	422	721	
**Size**			< 0.05
0-0.5 cm	32	80	
0.5-1 cm	144	466	
1-1.5 cm	89	120	
> 1.5 cm	157	55	
**Adjacent anterior capsule**			< 0.05
**<** 1 mm	216	160	
1-2 mm	128	159	
≥ 2 nun	78	402	
**Distance to the lower pole**			< 0.05
0-10 mm	157	128	
10-20 mm	190	273	
≥ 20 mm	75	320	
**CDFI**			< 0.05
2-3	150	180	
0-1	272	541	
**Age(year)**			0.24
≥ 55	73	145	
< 55	349	576	
**A/T**			0.94
**> 1**	197	335	
≥ 1	225	386	
**Location**			0.19
left lobe	190	353	
right lobe	208	341	
Isthmic	24	27	
**Adjacent posterior capsule**			0.68
**<** 1 mm	104	162	
1-2 mm	88	150	
≥ 2 mm	230	409	
**Distance to the upper pole**			0.26
0-10 mm	25	61	
10-20 mm	176	283	
≥ 20 mm	221	377	
**Microcalcification**			0.17
Present	325	529	
Absent	97	192	
**Smooth border**			0.14
Absent	358	634	
Present	64	87	
**Ultrasonic halo**			0.18
Absent	406	681	
Present	16	40	
**Hypoechoic**			0.63
Present	405	696	
Absent	17	25	
**Hashimoto's thyroiditis**			0.62
Present	120	215	
Absent	302	506	

**Table 3:** Multivariate logistic regression analysis of risk factors for male.

Characteristics	β	Odds ratio [95% CI]	*p*
**Male size**			
0.5-1 cm	2.62	1.18-5.81	<0.05
1-1.5 cm	6.93	3.01-15.95	<0.05
> 1.5 cm	12.12	5.21-28.18	<0.05
**Adjacent anterior capsule**			
1-2 mm	2	1.32-3.03	<0.05
**< 1 mm**	2.81	1.83-4.33	<0.05
**Distance to the lower pole**			
10-20 mm	1.52	1.02-2.27	<0.05
0-10 mm	2.63	1.69-4.09	<0.05
**CDFI**			
2-3	0.69	0.48-1.00	<0. 05

**Table 4:** Multivariate logistic regression analysis of risk factors for female.

Characteristics	β	Odds ratio[95% CI]	*p*
**Female**			
**size**			
0.5-1 cm	0.89	0.53-1.49	<0.05
1-1.5 cm	1.43	0.81-2.52	<0.05
> 1.5 cm	4.9	2.73-8.77	<0.05
**Adjacent anterior capsule**			
1-2 mm	3.21	2.21-4.66	<0.05
< 1 mm	5.14	3.60-7.34	<0.05
**Distance to the lower pole**			
10-20 mm	2.06	1.44-2.94	<0.05
0-10 mm	3.58	2.36-5.42	<0.05
**CDFI**			
2-3	1.82	1.33-2.48	<0. 05

## Discussion

In the present study, we found and validated several US-based characteristics for predicting the probability of CLNM in cN0 unifocal PTC patients. The patients in the present study met the following criteria based on preoperative detection: the suspected nodule was confirmed to be PTC by biopsy; the nodule was unifocal and less than 4 cm in diameter; no prior neck radiation exposure; no extrathyroidal extension; no CLNM; and no distant metastases. The present findings indicated that these risk factors may improve the preoperative prediction of CLNM in a noninvasive manner. The sensitivity for detecting CLNM using preoperative neck US imaging is low (31, 32) due to air in the trachea, complex structures in the sternum and clavicle, which make it difficult for US imaging to detect CLNM.

For clinically N0 unifocal PTCs, the precise evaluation of CLNM is important. Barczynski et al. acknowledged that CLND promotes both a locoregional situation and 10-year disease-specific survival without increasing the risk of permanent morbidity (33). Hartl et al. reported that CLND does not enhance the incidence of morbidity, especially the permanent dissections (34), which may be due to the surgical skills of the surgeons reducing complications. In our study, all of the complications and side effects were documented and treated. The complications of surgery included hypocalcemia, hoarseness, seroma, pain and choke. The complications were totally under control and there were no permanent injury caused. It is well-known that revision surgery in scarred areas promotes a high risk for recurrent laryngeal nerve (RLN) injury and parathyroid gland injury. Zhao et al. indicated that CLND significantly lowers LN recurrence (35). In addition, CLND helps surgeons assess the tumor-node-metastasis (TNM) stage of patients with PTC to determine the subsequent radioactive iodine (RAI) therapy (36). However, ATA and NCCN guidelines do not recommend prophylactic CLND. Nixon et al. found that the 5- and 10-year disease-free survival of patients with PTC who did not undergo prophylactic CLND is 100%; they considered an active observation of CLN safe and that it should be suggested for all patients with PTC considered before and during surgery without central neck metastasis (37). Furthermore, many researchers have suggested that prophylactic CLND may promote the complication rate of RLN and parathyroid gland permanent injury by approximately 2-fold (38, 39). Above all, CLND is important but should be implemented with care. Further, it is imperative to diagnose CLNM preoperatively for clinical decision-making processes.

In the present study, an US feature was generated using risk factors, including tumor size, for the prediction of CLNM. In the present study, tumors with a larger size on US examination were more likely to be related to CLNM, which was consistent with other reports (40). Tumor size is widely analyzed in many staging systems, including the American Joint Committee on Cancer (AJCC) staging system. The most used cutoff in risk stratification is 1 cm, which is widely accepted as a risk factor for CLNM and is associated with higher mortality (41). Many studies have utilized the largest diameter of the tumor as the tumor size, but there is no definitive conclusion at present (42). However, some studies have reported that tumor size is not an adequate independent predictor of CLNM (43). Several previous studies have set the size threshold between 5 and 10 mm; however, these studies have reported that when the tumor is less than this threshold, the rate of CLNM is still high, ranging from 26% to 55% (44, 45).

The location features of the nodule may also be important risk factors. A tumor adjacent to the anterior capsule and that has a short distance to the lower pole has a close association with CLNM (46). The thyroid gland is encapsulated by a thin fibroelastic (true) capsule, and this capsule is covered by a pretracheal fascia from the outside and is called a false capsule. The true capsule gives rise to septa deep into the parenchyma, dividing the thyroid gland into lobules. The septa makes room for blood vessels, nerves and lymphatics in the gland. The thyroid gland and its neighboring structures have many lymphatics, which drain the thyroid in almost every direction. Within the thyroid gland, lymphatic channels are present beneath the capsule and connect lobes through the isthmus. Most thyroid neoplasms drain directly to CLN basins, except for cancers in the superior third of the gland, which may drain to the lateral compartment (known as skip metastases) (47). This may be the reason for the association of a closer distance to the capsule and lower pole on US imaging with CLNM. In the present study, the CDFI was significantly different between the CLNM-positive and CLNM-negative groups; richer blood supply was correlated with a higher probability of CLNM (48).

The present study had several limitations, including those inherent to a retrospective study design. The present study was also a single-center historical cohort study, and our results may have been biased accordingly. Stringent external validation needs to be performed in larger, prospective multicenter clinical trials to obtain a more objective conclusion. In addition, a relatively small number of patients had large-volume CLNM, which did not allow us to demonstrate the key predictive factor of occult large-volume CLNM. The performance of our prediction depends on the accuracy of operator-reported imaging characteristics. The criteria used to evaluate the US features were subjective. However, the interobserver agreement for each feature in the present study was good. Although we did not evaluate the recurrence of PTC according to different clinical factors, our findings are still important for clinicians to make decisions on management strategies for cN0 unifocal PTC. The CLNM status is an indicator of aggressive behavior in PTC, but its evaluation has been limited in imaging studies. The present study suggested that the size, adjacent anterior capsule, distance to the lower pole and CDFI of cN0 unifocal PTC patients are good preoperative clinical factors that predict the occult CLNM status. Thus, it may be appropriate to perform more precise inspection or surgical intervention rather than active surveillance for those patients.

In summary, the present study revealed several risk factors based on US imaging characteristics, suggesting that this easy-to-use method can be applied to facilitate preoperative individualized prediction of occult CLNM in cN0 unifocal PTC patients, which is in line with the current trend towards precision medicine.

## Data Availability Statement

The raw data supporting the conclusions of this article will be made available by the authors, without undue reservation.

## Ethics Statement

The studies involving human participants were reviewed and approved by the Ethics Committee of the Guangdong Provincial People’s Hospital, Guangdong Academy of Medical Sciences (Guangzhou, Guangdong Province, People’s Republic of China). Written informed consent was not required for this study, in accordance with the local legislation and institutional requirements.

## Author Contributions

YL, JH, and ZZ, have contributed equally to this work and share first authorship. They are responsible for research design, data collecting, analysis and writing. YH and JD are responsible for research design and data collecting. SW and ZW are responsible for research design, data analysis. All authors contributed to the article and approved the submitted version.

## Funding

This study was supported by the Natural Science Foundation of Guangdong Province (No. 2020A1515010127) and Guangdong Provincial People’s Hospital Scientific Foundation for Distinguished Young Scholars of Guangdong Province (No. KJ012019441).

## Conflict of Interest

The authors declare that the research was conducted in the absence of any commercial or financial relationships that could be construed as a potential conflict of interest.

## Publisher’s Note

All claims expressed in this article are solely those of the authors and do not necessarily represent those of their affiliated organizations, or those of the publisher, the editors and the reviewers. Any product that may be evaluated in this article, or claim that may be made by its manufacturer, is not guaranteed or endorsed by the publisher.
